# The Tonsils Revisited: Review of the Anatomical Localization and Histological Characteristics of the Tonsils of Domestic and Laboratory Animals

**DOI:** 10.1155/2011/472460

**Published:** 2011-08-21

**Authors:** Christophe Casteleyn, Sofie Breugelmans, Paul Simoens, Wim Van den Broeck

**Affiliations:** Department of Morphology, Faculty of Veterinary Medicine, Ghent University, Salisburylaan 133, 9820 Merelbeke, Belgium

## Abstract

This paper gives an overview of the anatomical localization and histological characteristics of the tonsils that are present in ten conventional domestic animal species, including the sheep, goat, ox, pig, horse, dog, cat, rabbit, rat, and pigeon. Anatomical macrographs and histological images of the tonsils are shown. Six tonsils can be present in domestic animals, that is, the lingual, palatine, paraepiglottic, pharyngeal, and tubal tonsils and the tonsil of the soft palate. Only in the sheep and goat, all six tonsils are present. Proper tonsils are absent in the rat, and pigeon. In the rabbit, only the palatine tonsils can be noticed, whereas the pig does not present palatine tonsils. The paraepiglottic tonsils lack in the ox, horse, and dog. In addition, the dog and cat are devoid of the tubal tonsil and the tonsil of the soft palate.

## 1. Introduction

An animal's body is connected with the external environment through the skin and mucosae. As a result, these lining tissues are exposed to many hazards, amongst which foreign antigens play a major immunological role. Compared to the skin, the mucosae represent the weaker barrier. To protect the body, a well-equipped defence mechanism is present in the skin and along many mucosal linings: the SALT (skin-associated lymphoid tissue) and MALT (mucosa-associated lymphoid tissue), respectively [1–3].

According to the anatomical and physiological characteristics of the different mucosae, MALT can further be specified into gut-associated lymphoid tissue (GALT), gastric-MALT, nasal-cavity-associated lymphoid tissue (NALT), larynx-associated lymphoid tissue (LALT), trachea-associated lymphoid tissue (TALT), bronchus-associated lymphoid tissue (BALT), conjunctiva-associated lymphoid tissue (CALT), (salivary) duct-associated lymphoid tissue (DALT), lacrimal duct-associated lymphoid tissue (LDALT), and vascular-associated lymphoid tissue (VALT) [4]. Apart from these MALT sites, aggregations of lymphoid cells are present in the lamina propria mucosae of many other tissues such as the genital tract [5, 6].

Although the various components of MALT are anatomically separated from each other, they are functionally connected through the so-called common mucosal immune system [7]. As such, through lymphocyte recirculation, immune responses initiated in one of the inductive sites of MALT can exert immunity at many effector sites, including the genital tract and the mammary and salivary glands [8–12]. T- and B-cell activation at one of the MALT sites can thus result in T-cell responses and the secretion of immunoglobulins at distant effector sites [7, 9, 13–15].

The tonsils are major components of the MALT. They consist of aggregations of lymphoid cells that are present in the mucosa of the nasopharynx (NALT), the oropharynx (GALT), and the laryngopharynx (LALT) [16]. As a result, all tonsils together form a ring of lymphoid tissue in the pharyngeal wall, called the “Waldeyer ring” [17–19]. Its location at the crossing of the digestive and respiratory tracts plays a key role in immunity as this is the site where vast amounts of foreign antigens enter the body during feeding and breathing [20, 21].

Due to their role in immune responses and in the pathogenesis of several diseases including prion diseases, tonsils have gained much scientific interest during the past decade. Many studies have thoroughly described the immunological characteristics of the tonsils of various mammals, and special attention has been paid to the mechanisms of antigen uptake. However, the larger amount of immunological investigations are limited to those tonsils that are most easily identified, such as the palatine tonsils. The study of other tonsils seems to be hampered by the small amount of basic anatomical and histological data present in the literature. Moreover, available data are scattered in conventional anatomical textbooks, and some inconsistencies between several sources can be noticed. Therefore, this paper offers an overview of the anatomical localization and the histological characteristics of the tonsils that are present in ten conventional domestic animal species.

## 2. Materials and Methods

Heads of sheep (*n* = 6), goat (*n* = 6), cattle (*n* = 3), pig (*n* = 3), horse (*n* = 2), dog (*n* = 4), cat (*n* = 4), rabbit (*n* = 3), rat (*n* = 3), and pigeon (*n* = 3) were obtained after euthanasia of animals that had been used in other studies. The localization of the tonsils was demonstrated by anatomical dissection. To visualize lymphoid tissue that is not macroscopically visible on fresh tissues, the heads were fixed in 2% acetic acid for 4 h after which lymphoid tissue colours white [22]. Photographs were taken using a digital camera (Canon EOS 300D, Diegem, Belgium). Subsequently, samples for histological analysis were collected. These were fixed in 3.5% buffered formaldehyde at room temperature for three days, dehydrated in a tissue processor (Microm STP420D, Prosan, Merelbeke, Belgium), and embedded in paraffin using an embedding station (Microm EC350-1 and 350-2, Prosan). Tissue sections (8 *μ*m thick) were made (Microm HM360 microtome, Prosan) from all blocks, mounted on slides, stained with hematoxylin (Haematoxylin (C.I. 75290), Merck KGaA) and eosin (Eosine yellow (C.I. 45380), VWR international bvba/sprl), and examined with a motorized microscope (Olympus BX61, Olympus Belgium) linked to a digital camera (Olympus DP50, Olympus Belgium).

## 3. Results

### 3.1. Sheep (Figure 1)

Six tonsils can be observed in the sheep, that is, the lingual tonsil (*tonsilla lingualis*), the palatine tonsil (*tonsilla palatina*), the paraepiglottic tonsil (*tonsilla paraepiglottica*), the pharyngeal tonsil (*tonsilla pharyngea*), the tubal tonsil (*tonsilla tubaria*), and the tonsil of the soft palate (*tonsilla veli palatini*).

The oropharyngeal *lingual tonsil* is not macroscopically visible since it consists of small aggregations of lymphoid cells that are mainly present within the connective tissue cores of the vallate gustatory papillae. These are located at the lateral sides of the root of the tongue. Small lymphoid cell aggregations or scattered lymphoid cells are also located in between the lingual muscles and the salivary glands in the region of the vallate papillae. The lingual tonsil is entirely covered by a keratinized stratified squamous epithelium.

The *palatine tonsil* is bilaterally present in the oropharynx and is located between the palatoglossal and palatopharyngeal arches. It is an ovoid structure, the size of a hazelnut, that contains one to three narrow elongated entrances (*fossulae tonsillares*) to the underlying crypts (*cryptae tonsillares*) which have a few diverticula. Each crypt lies in the centre of a tonsillar follicle (*folliculus tonsillaris*) that contains lymphoid tissue composed of numerous, mainly secondary, lymphoid follicles (*lymphonodulus*) divided by interfollicular regions. Each tonsillar follicle is surrounded by a capsule of connective tissue. Within the crypts, the overlying nonkeratinized stratified squamous epithelium is irregularly modified into a reticular epithelium due to heavy infiltration by lymphoid cells. 

The bilaterally present *paraepiglottic tonsil* is located in the laryngopharynx lateral to the base of the epiglottis. It is macroscopically visible as a few nodular mucosal elevations that are separated by deep invaginations. Dense aggregations of lymphoid cells and primary and secondary lymphoid follicles that are separated by interfollicular regions are present within the mucosal elevations. The lining epithelium is stratified squamous and slightly keratinized. Lymphoid cell infiltration can be present in the epithelium directly overlying secondary lymphoid follicles.

The *tonsil of the soft palate* is located at the dorsal surface (nasopharyngeal side) of the soft palate, but is only macroscopically visible after 4 h fixation in 2% acetic acid, after which white, scattered nodules appear. The rostral part of the tonsil consists of a continuous layer of lymphoid cells separated from the respiratory epithelium by a thin layer of connective tissue. Primary and secondary lymphoid follicles, which are covered by a thin stratified squamous epithelium, are seen in the middle part. Towards the caudal edge of the soft palate, only scattered small aggregations of lymphoid cells are present.

The prominent *pharyngeal tonsil* is located dorsally in the nasopharynx on the caudal part of the pharyngeal septum. It measures approximately 2-3 cm in length and 1 cm in width and height. Numerous folds, mainly longitudinally arranged, are present on its surface. The lymphoid tissue surrounds a connective tissue core rich in blood and lymph vessels and is organized into secondary lymphoid follicles with interfollicular regions. At many places, the covering pseudostratified columnar ciliated epithelium is invaded by varying amounts of lymphoid cells resulting in the formation of randomly distributed patches of reticular nonciliated epithelium.

The *tubal tonsil* is bilaterally situated in the lateral nasopharyngeal wall around and caudoventral to the opening of the auditory tube. It is, however, only macroscopically visible after 4 h fixation in 2% acetic acid, after which white, scattered nodules appear. On histology, the lymphoid tissue is scattered and varies from aggregated lymphoid cells to primary and secondary lymphoid follicles. In areas overlying the lymphoid follicles, the pseudostratified columnar ciliated epithelium is often infiltrated by lymphoid cells and is characterized by a lack of cilia.

### 3.2. Goat (Figure 2)

Like in sheep, all six tonsils are present in the goat. The localization and volume of the caprine tonsils are similar to that of the ovine tonsils.

The *lingual tonsil* consists of aggregated lymphoid cells that are mainly present within the vallate papillae. Small lymphoid cell aggregations are also located just underneath the keratinized stratified squamous epithelium of the lingual root, in between the lingual muscles and the salivary glands in the region of the vallate papillae.

The *palatine tonsil* is larger than that in sheep, and its crypt system is more extensive. A few crypt openings, which lead to several (up to five) tonsillar follicles, are macroscopically visible in the lateral oropharyngeal wall. Large diverticles originate from the crypts. The lymphoid tissue around the crypts contains many secondary lymphoid follicles separated by interfollicular lymphoid tissue. Just like in sheep, the overlying nonkeratinized stratified squamous epithelium within the crypts is irregularly modified into a reticular epithelium.

The *paraepiglottic tonsil* can only be found in the minority of animals using histology. When present, it is macroscopically hardly visible as a small mucosal elevation which contains primary and secondary lymphoid follicles that are separated by interfollicular regions. The lining epithelium is stratified, squamous, and slightly keratinized, and it can contain some infiltrating lymphoid cells.

The *tonsil of the soft palate* is less prominent compared to sheep and is also not macroscopically visible on fresh tissue. Histologically, it is represented by a few isolated primary or secondary lymphoid follicles just underneath the lining epithelium which is pseudostratified, columnar, and ciliated on the rostral half of the soft palate and stratified squamous on the caudal half.

The *pharyngeal tonsil* presents many infolds on its surface. These show numerous secondary folds that form crypts within the lymphoid tissue. The latter consists of secondary lymphoid follicles and interfollicular lymphoid tissue and surrounds a connective tissue core rich in blood and lymph vessels. At many places, the covering pseudostratified columnar ciliated epithelium is infiltrated by lymphoid cells resulting in the formation of patches of reticular epithelium.

The *tubal tonsil* is not macroscopically visible on fresh tissue. Around and caudoventral to the opening of the auditory tube, primary and secondary lymphoid follicles and diffuse lymphoid tissue containing scattered lymphoid cells can be observed on histological examination. The lining pseudostratified columnar ciliated epithelium is sometimes invaded by lymphoid cells.

### 3.3. Ox (Figure 3)

Five tonsils are present in the ox, that is, the lingual tonsil, the palatine tonsil, the pharyngeal tonsil, the tubal tonsil, and the tonsil of the soft palate. The paraepiglottic tonsil lacks. The location of the present bovine tonsils is the same as in the small ruminants.

The *lingual tonsil *is very well developed in the ox. It is macroscopically visible as rostrolaterally orientated rows of tonsillar fossules at the root of the tongue, caudal to the vallate papillae. These fossules represent the crypt openings of tonsillar follicles that are surrounded by aggregations of secondary lymphoid follicles with interfollicular regions. The keratinized stratified squamous epithelium of the crypts is often infiltrated by lymphoid cells. Several aggregated lymphoid follicles are also present in between the tonsillar follicles just underneath the epithelium. In addition, some primary lymphoid follicles and aggregations of lymphoid cells are located in the subepithelial connective tissue layer which extends from 2 cm caudal to 3 cm rostral to the most caudal vallate papillae.

The large ovoid *palatine tonsil* (4 to 5 cm in diameter) is bilobated and contains a central cavity (*sinus tonsillaris*) of which the entrance is visible at the oropharyngeal surface. Many tonsillar fossules open into this tonsillar sinus and lead to numerous crypts that lie in the centres of tonsillar follicles. The lymphoid tissue surrounding the crypts is composed of many secondary lymphoid follicles with interfollicular lymphoid tissue. The crypt epithelium is stratified squamous and often infiltrated by lymphoid cells.

The large *pharyngeal tonsil *is approximately 3-4 cm long and shows many epithelial infolds that sometimes form crypts. The lymphoid tissue, consisting mainly of secondary lymphoid follicles separated by interfollicular lymphoid tissue, is organized around a central connective tissue core. Like in the small ruminants, the covering pseudostratified columnar ciliated epithelium is often reticulated.

The *tubal tonsil *is macroscopically visible as a cluster of irregular mucosal elevations caudoventral to the opening of the auditory tube. It consists of primary and secondary lymphoid follicles that are separated by interfollicular lymphoid tissue. The tonsil is lined by a pseudostratified columnar ciliated epithelium that is often infiltrated by lymphoid cells where lymphoid follicles are covered.

The *tonsil of the soft palate *is a diffusely organized tonsil located at the nasopharyngeal side of the soft palate just underneath the respiratory epithelium. It consists of scattered primary and secondary lymphoid follicles. Interfollicular lymphoid tissue is not abundant. Some lymphoid cell infiltration of the epithelium overlying lymphoid follicles can be present.

### 3.4. Pig (Figure 4)

Five tonsils are present in the pig, that is, the lingual tonsil, the paraepiglottic tonsil, the pharyngeal tonsil, the tubal tonsil, and the tonsil of the soft palate. The palatine tonsil is absent.

The *lingual tonsil *is more prominent in pigs compared to small ruminants, but less developed compared to the ox. It consists of encapsulated lymphoid clusters containing mainly secondary lymphoid follicles separated by interfollicular lymphoid tissue. These clusters are located at the bases of and within the mechanical papillae of the root of the tongue (*papillae tonsillares*), caudal to the gustatory vallate and foliate papillae. Deep crypts are formed between adjacent tonsillar papillae which are lined by a slightly keratinized stratified squamous epithelium. Lymphoid cell infiltration can sometimes be observed within the crypt epithelium. 

The *paraepiglottic tonsil *is located craniolateral to the base of the epiglottis. It is macroscopically present as a round to oval plaque of approximately 1-2 cm in diameter which possesses a few tonsillar fossules. The lymphoid tissue surrounding the crypts is organized mainly into secondary lymphoid follicles separated by interfollicular lymphoid tissue and forms tonsillar follicles. Lymphoid cell infiltration of the stratified squamous crypt epithelium is possible.

The *pharyngeal tonsil *of the pig is macroscopically visible in the roof of the nasopharynx as a prominence of the caudal end of the pharyngeal septum. It is composed of lymphoid tissue that is macroscopically organized into longitudinal mucosal folds. This tissue consists of encapsulated clusters of mainly secondary lymphoid follicles and interfollicular lymphoid tissue. Tonsillar follicles are often formed by invaginations of the lining respiratory epithelium. Lymphoid cells can be present within the crypt epithelium.

The *tubal tonsil *can be noticed macroscopically by the irregular nasopharyngeal mucosal surface caudoventral to the orifice of the auditory tube. The lymphoid tissue is mainly composed of secondary lymphoid follicles with interfollicular regions and is often organized into tonsillar follicles with a central crypt. This crypt is lined by a respiratory epithelium in which lymphoid cells can reside.

The *tonsil of the soft palate *is especially well developed in the pig. It is present as a bilateral oval plaque of lymphoid tissue, approximately 5 cm in length and 3 cm in width. Numerous tonsillar fossules are visible at the ventral side of the soft palate. These lead to crypts that are located in the centres of the tonsillar follicles which are composed of secondary lymphoid follicles that are separated by diffuse lymphoid tissue. The oropharyngeal epithelium covering the tonsil is slightly keratinized, stratified, and squamous and is often infiltrated by lymphoid cells at the level of the crypts.

### 3.5. Horse (Figure 5)

Five tonsils are present in the horse, that is, the lingual tonsil, the paired palatine tonsil, the pharyngeal tonsil, the paired tubal tonsil, and the tonsil of the soft palate. The paraepiglottic tonsil is not present in the horse.

The *lingual tonsil *is well developed. It can be identified macroscopically by the presence of many tonsillar fossules located at the dorsolateral sides of the root of the tongue. Each fossule leads to a crypt that is surrounded by lymphoid tissue mainly composed of secondary lymphoid follicles and interfollicular regions. The tonsillar follicles formed by this arrangement are well encapsulated. The epithelium covering the tongue is stratified, squamous, and keratinized. Within the tonsillar follicles, the crypt epithelia are infiltrated by lymphoid cells at many places.

The *palatine tonsil *is an elongated flat structure which lies bilaterally on the floor of the oropharynx, caudolateral to the tongue. It is approximately 10 cm long and 2 cm wide. Many tonsillar fossules leading to crypts are visible at the surface. Around the crypts, mainly secondary lymphoid follicles with interfollicular lymphoid tissue are present and form tonsillar follicles. The slightly keratinized stratified squamous epithelium can be transformed into a reticular epithelium at the level of the crypts.

The *pharyngeal tonsil *is not well delineated and macroscopically hardly visible in the horse. It is diffusely present in the nasopharyngeal wall, and the pharyngeal recess as primary and secondary lymphoid follicles and nonorganized lymphoid tissue. The overlying respiratory epithelium is slightly folded and sometimes infiltrated by lymphoid cells.

The *tubal tonsil *is located near the opening of the auditory tube, but is continuous with the pharyngeal tonsil. Since the border between both tonsils is not clear in this species, it is impossible to exactly delineate both tonsils. The tubal tonsil has many characteristics in common with the pharyngeal tonsil. The lymphoid tissue is, however, less abundant and more diffuse.

The *tonsil of the soft palate *is prominent. Macroscopically, a slightly raised, oval area (3-4 cm long and 2-3 cm wide) with many tonsillar fossules is located centrally at the ventral side of the soft palate. Tonsillar follicles with a central crypt surrounded by primary and secondary lymphoid follicles and interfollicular lymphoid tissue can be seen, but aggregations of lymphoid follicles and diffuse lymphoid tissue are also present. The lining epithelium is slightly keratinized and stratified squamous. Lymphoid cell infiltration of the epithelium is common.

### 3.6. Dog (Figure 6)

Three tonsils are present in the dog, that is, the lingual tonsil, the paired palatine tonsil, and the pharyngeal tonsil.

The *lingual tonsil *is small in dogs. Diffuse lymphoid tissue consisting of disseminated aggregations of lymphoid cells is located at the base of the tongue. The keratinized stratified squamous epithelium can be infiltrated by lymphoid cells in regions overlying the lymphoid tissue.

The reddish *palatine tonsil *is located in the lateral sides of the oropharynx between the palatoglossal and palatopharyngeal arches. A tonsillar fossa (*fossa tonsillaris*) is formed by the presence of a crescent mucosal fold (*plica semilunaris*) from the ventral surface of the lateral part of the soft palate. It forms the medial wall of the fossa. The lymphoid tissue is composed of primary and secondary lymphoid follicles with interfollicular regions. The overlying stratified squamous epithelium is thin and massively infiltrated by lymphoid cells.

The *pharyngeal tonsil *is not prominently present in the nasopharynx, dorsal to the openings of the auditory tubes. Its surface is smooth and shows no tonsillar fossules. It contains flattened aggregations of lymphoid cells and primary and secondary lymphoid follicles. The overlying epithelium is pseudostratified, columnar, and ciliated and seldom infiltrated by lymphoid cells. 

### 3.7. Cat (Figure 7)

Four tonsils are present in the cat, that is, the lingual tonsil, the paired palatine tonsil, the paired paraepiglottic tonsil, and the pharyngeal tonsil.

The *lingual tonsil *is poorly developed and cannot be observed macroscopically. It consists of aggregations of lymphoid cells located in the mucosa of the root of the tongue. No infiltration of lymphoid cells into the overlying keratinized squamous epithelium is present.

The reddish *palatine tonsil *is proportionally larger in the cat compared to the dog since the semilunar fold also contains lymphoid tissue composed of mainly secondary lymphoid follicles and interfollicular regions. As such, the tonsil has an oval shape on a transverse section. The tonsillar fossa leads to a central tonsillar sinus lined by keratinized squamous epithelium which is often heavily infiltrated by lymphoid cells.

The *paraepiglottic tonsil *is located at the same position as in the sheep and pig. The complexity of the tonsil varies between individuals. Well-developed tonsils can be identified macroscopically by a few mucosal elevations which contain primary and secondary lymphoid follicles. Less manifest tonsils only present aggregations of lymphoid cells and are macroscopically not visible. The thin lining epithelium is stratified squamous and heavily infiltrated by lymphoid cells when in contact with the underlying lymphoid tissue.

The *pharyngeal tonsil *is located in the roof of the nasopharynx dorsal to the orifices of the auditory tubes. Its macroscopic appearance and histological characteristics are very similar to those of the dog.

### 3.8. Rabbit (Figure 8)

Only the *palatine tonsil* is present in the rabbit. It can be recognized as a bilateral oval protrusion of the dorsolateral wall of the oropharynx. Upon visual inspection, a crescent tonsillar fossa surrounded by a thick semilunar fold is obvious. This fold and the tonsil itself are lined by keratinized squamous epithelium that is transformed into a reticular epithelium at many places. The palatine tonsil harbours multiple secondary lymphoid follicles which are separated by diffuse interfollicular tissue. 

No tonsils are present in the nasopharynx. However, aggregations of lymphoid tissue and lymphoid follicles can be observed histologically at the bottom of the ventral nasal meatus and the nasopharyngeal meatus. In addition, isolated lymphoid follicles are present within the mucosa of the nasal conchae and the lateral walls of the nasal cavity. This lymphoid tissue is called nasal-cavity-associated lymphoid tissue (NALT). The respiratory epithelium overlying the NALT can be infiltrated by lymphoid cells.

### 3.9. Rat (Figure 9)

The rodents that were examined in the present study lacked any tonsils. Instead, well-developed NALT was present. It was located at the bottom of the ventral nasal meatus and the nasopharyngeal meatus. It resembled the NALT of the rabbit histologically.

### 3.10. Pigeon (Figure 10)

Lymphoid tissue is located at the oral and nasal sides of the palate, around the choanal and infundibular clefts, rostral to the pharyngeal papillae. This lymphoid tissue can be designated as pharyngeal tonsil. The lymphoid tissue can be present as aggregations of lymphoid cells or be organized into primary and secondary lymphoid follicles. Interfollicular lymphoid tissue is not abundant. Infiltration of the keratinized stratified squamous epithelium lining the oral side of the palate and the respiratory epithelium lining the nasal side is very limited.

The presence and relative volume of the tonsils and lymphoid tissues in the various domestic animals examined in this study are comparatively listed in Table 1.

## 4. Discussion

The number of studies investigating the immune functions of the tonsils have increased substantially since it was acknowledged during the past decade that tonsils form a first line of defence against foreign antigens or play a role in the propagation of infectious diseases. As a result, more and more researchers entering the field of mucosal immunology are interested in the anatomical localizations and the morphology of these MALT structures. Unfortunately, morphological data of the tonsils are scattered in conventional anatomical textbooks. Moreover, inconsistencies between several sources can be noticed. Therefore, the present paper offers basic morphological information of the tonsils of ten conventional domestic and laboratory animal species, describing their localization and histological characteristics.

The inconsistencies that are present in the literature mainly deal with the anatomical localization and the nomenclature of the tonsils [23]. Tonsils are *lymphonoduli aggregati *which means that they consist of an aggregation of lymph nodules (*lymphonodulus* or *nodulus lymphaticus*) [24]. Lymph nodules are, however, more commonly known in the immunological literature as lymphoid follicles. As a result, some presumed tonsils, such as the lingual tonsils in small ruminants which does not contain lymphoid follicles but only small aggregations of lymphoid cells, cannot be considered as proper tonsils [25, 26]. 

Since follicles contain a lumen, this term is not appropriate to define a well-circumscribed aggregation of lymphoid cells [24, 27]. Instead, the term tonsillar follicle is used to define the tonsillar structure that is composed of a crypt, its orifice (*fossula*), and its surrounding lymphoid tissue [24]. Tonsillar follicles are, for example, typical in the palatine tonsils of ruminants. The term lymphoid follicle is, however, preferred above the term lymph nodule in the immunological literature [28]. Therefore, this terminology is also applied in the present paper. The other terms used here derive from the official list of veterinary anatomical nomenclature [24].

The most prominent ambiguity concerning the localization of the tonsils can be noticed when studying the literature about the tonsil of the soft palate. The present study, together with our previous reports [16, 28, 29], unequivocally shows that this tonsil is located at the nasopharyngeal side of the soft palate in ruminants. In conventional anatomical textbooks; however, the tonsil of the soft palate is allocated to the oropharyngeal side of the soft palate in these species [30, 31]. In the pig and horse, the well-developed tonsil of the soft palate is located at the oropharyngeal side of the soft palate. This tonsil is sometimes erroneously called the palatine tonsil in the pig [32]. However, the latter tonsil lacks in this species. Also in horses, the tonsil of the soft palate has been designated by other names such as *tonsilla palatina impar* or *tonsilla veli palatini impar* [32, 33]. In domestic carnivores, a tonsil of the soft palate that is located at the oropharyngeal side of this anatomical structure is mentioned by Schummer and Nickel [34] and Habermehl [35]. This tonsil was, however, not found in the present study nor was it observed in the dog by Cesta [7].

According to several authors, the presence of well-developed nasopharyngeal lymphoid tissue, and thus the location of the tonsil of the soft palate at the dorsal side of this structure, is an immunological adaptation to breathing through the nose which results in the transportation of many antigens along the nasopharyngeal mucosa [11, 12, 36]. This hypothesis was based on the observation that rodents, which breathe through the nose, lack any tonsils but possess a well-developed NALT in the nasopharynx [11, 12, 36]. However, the generalization of this assumption could be refuted since in cattle and horses, which also breathe through the nose, the oropharyngeal lymphoid tissue is well developed, and the tonsil of the soft palate is located at the oropharyngeal side of the soft palate in the horse. Moreover, the pharyngeal tonsil of horses, which cannot breathe through the mouth, is diffusely distributed compared to their massive lingual and palatine tonsils and their tonsil of the soft palate. The reason for this species difference is not obvious, but could, however, reflect an evolutionary advantage. 

In the present study, the complexity of the paraepiglottic tonsil varied in the cats examined. When no well-organized lymphoid tissue was present, the tonsil was macroscopically not visible. This may be the reason why some authors report that the paraepiglottic tonsil can be absent in the cat [32, 34, 37]. However, the histological examinations performed in this study have revealed that lymphoid tissue was present at the lateral bases of the epiglottis in each of the cats examined. In contrast, this study indicates that the paraepiglottic tonsil is inconstant in the goat, which means that it cannot be observed in each animal. This finding is not in accordance with the literature [33, 38, 39] that claims a constant presence of this tonsil in the caprine species. In sheep, all six tonsils, including the paraepiglottic tonsils, are always present. The variation in the development of the paraepiglottic tonsils could be a mere anatomical variation or be due to antigenic stimulation of the tonsillar tissue.

Besides their major immunological significance, tonsils can have clinical importance as well. Similar to humans, the palatine tonsils of the dog are prone to abscedation and recurrent tonsillitis [38]. In addition, prolapse of the palatine tonsils is possible in brachycephalic breeds as part of the brachycephalic airway obstruction syndrome [39, 40]. The latter pathology and the swelling of infected tonsils can result in respiratory distress which is an indication for tonsillectomy [38]. This surgical procedure can remarkably be performed without the risk of serious invasive infections later in life. A significant redundancy of tonsillar lymphoid tissue in the pharynx and the plasticity of the MALT in the lower respiratory and digestive tracts can be assigned as the underlying reasons [41, 42]. The continuous pharyngeal and tubal tonsils of the horse also have clinical relevance since chronic follicular pharyngitis or lymphoid follicular hyperplasia of the pharynx often occurs in young horses [38, 43, 44].

## 5. Conclusions

The tonsillar ring of the pharynx can be composed of up to six tonsils. The presence and location of these tonsils vary, however, according to the animal species. Large variations in histological characteristics exists between the several tonsils. Since the anatomical literature of the tonsils is sometimes ambiguous and basic knowledge of these structures is needed in immunological studies, the present paper could be valuable for both anatomists and immunologists. 

## Figures and Tables

**Figure 1:**
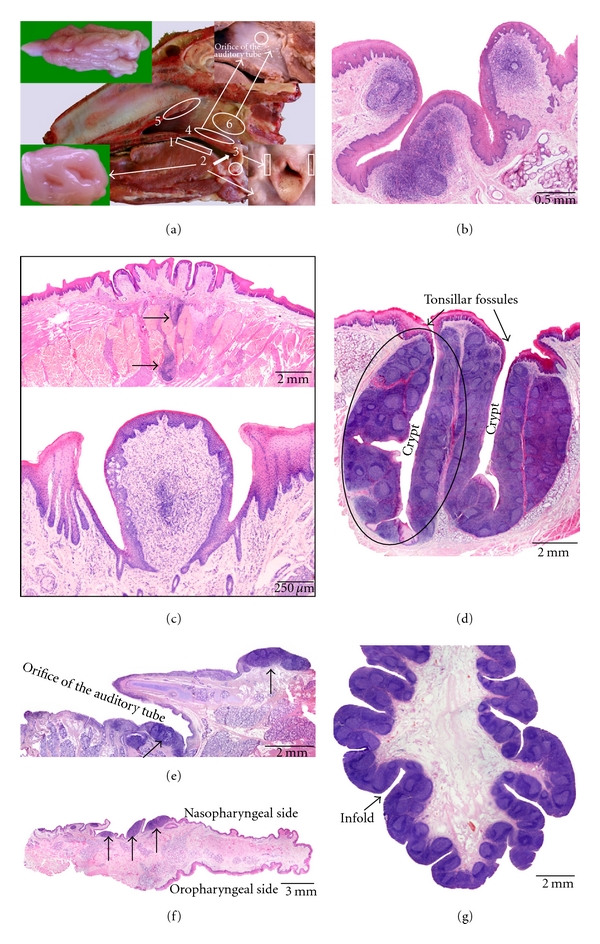
Anatomical localization and histological characteristics of the ovine tonsils. (a) Median section through an ovine head showing the location of the lingual tonsil (1), the palatine tonsil (2) of which a larger magnification is shown in the lower left insert, the paraepiglottic tonsil (3) that is located at the lateral basis of the epiglottis (lower right insert), the tonsil of the soft palate (4) consisting of scattered lymphoid nodules at the nasopharyngeal side of the soft palate (higher magnification after 2% acetic acid fixation for 4 h shown in the upper right insert), the pharyngeal tonsil (5) of which a larger magnification is presented in the upper left insert, and the tubal tonsil (6) consisting of scattered lymphoid nodules located caudoventral to the opening of the auditory tube (higher magnification after 2% acetic acid fixation for 4 h also shown in the upper right insert). (b) Histological section through the paraepiglottic tonsil. (c) Histological images of the lingual tonsil. The upper view shows aggregations of lymphoid cells (arrows) in between the lingual muscles and salivary glands. The lower image demonstrates the presence of an aggregation of lymphoid cells in the connective tissue core of a vallate papilla. (d–g) Histological sections through the palatine (d) and tubal (e) tonsils, the tonsil of the soft palate (f), and the pharyngeal tonsil (g), respectively. A tonsillar follicle is encircled in (d); the lymphoid tissue in (e) and (f) is indicated by the arrows.

**Figure 2:**
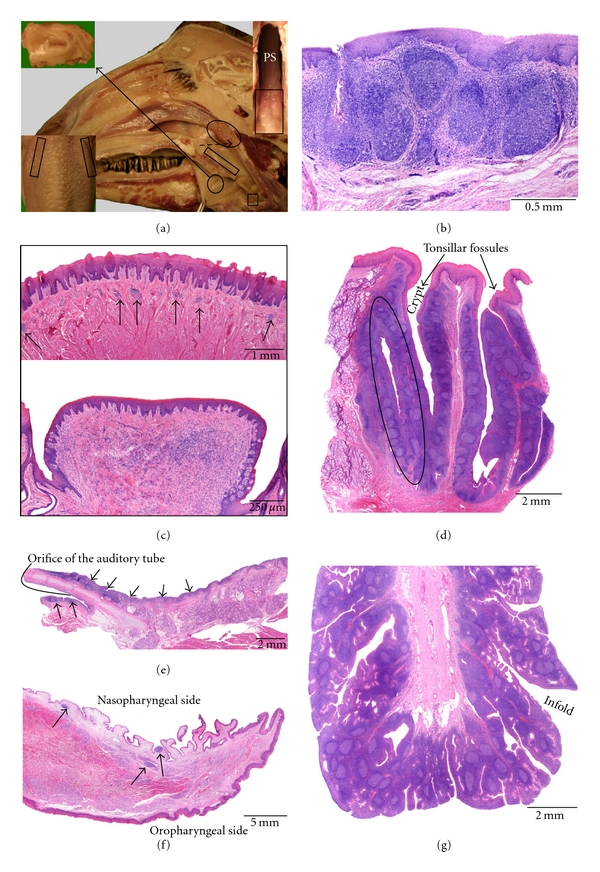
Anatomical localization and histological characteristics of the caprine tonsils. (a) Median section through a caprine head. A detail of the palatine tonsil is presented in the upper left insert. The location of the lingual tonsil, in the region of the vallate papillae, is shown in the lower left insert (dorsal view). The paraepiglottic tonsil that is located lateral to the basis of the epiglottis (small square box) is only present in a minority of goats. The position of the tonsil of the soft palate at the nasopharyngeal side of the soft palate is indicated by the rectangular box. At the caudal end of the pharyngeal septum (PS in the upper right insert), the pharyngeal tonsil (encircled on the overview, delineated by the rectangular box in the upper right insert) can be recognized by the numerous mucosal folds. The tubal tonsil is located caudoventral to the opening of the auditory tube, but is not visible on this view. (b) Histological section through the paraepiglottic tonsil. (c) Histological images of the lingual tonsil. The upper view shows aggregations of lymphoid cells (arrows) in between the lingual muscles and salivary glands. The lower image demonstrates the presence of an aggregation of lymphoid cells in the connective tissue core of a vallate papilla. (d–g) Histological sections through the palatine (d) and tubal (e) tonsils, the tonsil of the soft palate (f), and the pharyngeal tonsil (g), respectively. A tonsillar follicle is encircled in (d); the lymphoid tissue in (e) and (f) is indicated by the arrows.

**Figure 3:**
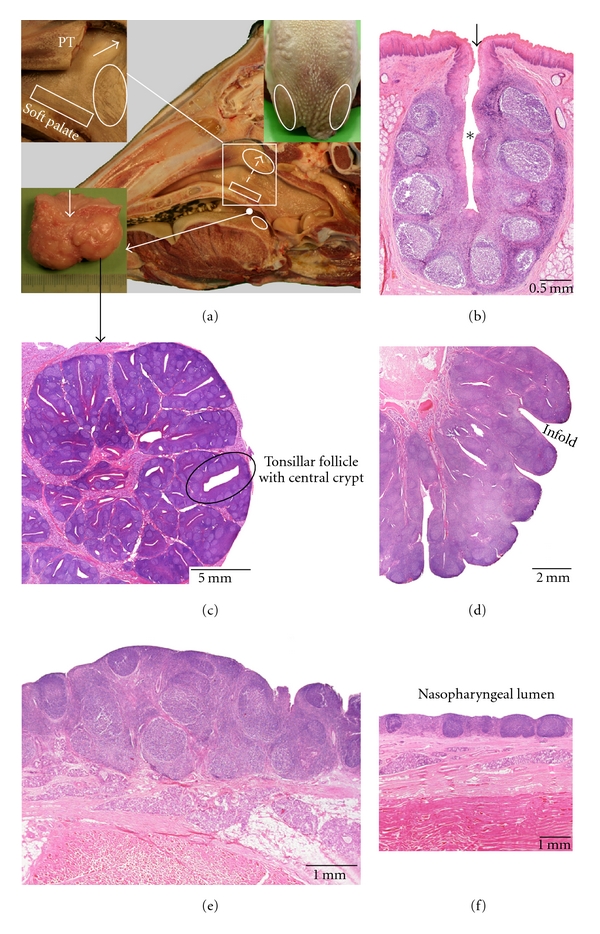
Anatomical localization and histological characteristics of the bovine tonsils. (a) Median section through a bovine head. In the upper left insert, the caudal part of the pharyngeal tonsil (PT, encircled in the central image) has been partly removed to demonstrate the tubal tonsil (encircled in the insert, indicated by the interrupted arrow in the central image) caudoventral to the opening of the auditory tube (arrow in the insert). The tonsil of the soft palate (boxed area) is located at the nasopharyngeal side of the soft palate. The bilobated palatine tonsil is shown in the lower left insert. Its sinus is indicated by the arrow. The position of the lingual tonsil at the root of the tongue is presented in the upper right insert. (b) Histological section through a tonsillar follicle belonging to the lingual tonsil. The tonsillar fossula and crypt are indicated by the arrow and the asterisk, respectively. (c–f) Histological sections through parts of the palatine (c), pharyngeal (d) and tubal (e) tonsils, and the tonsil of the soft palate (f), respectively.

**Figure 4:**
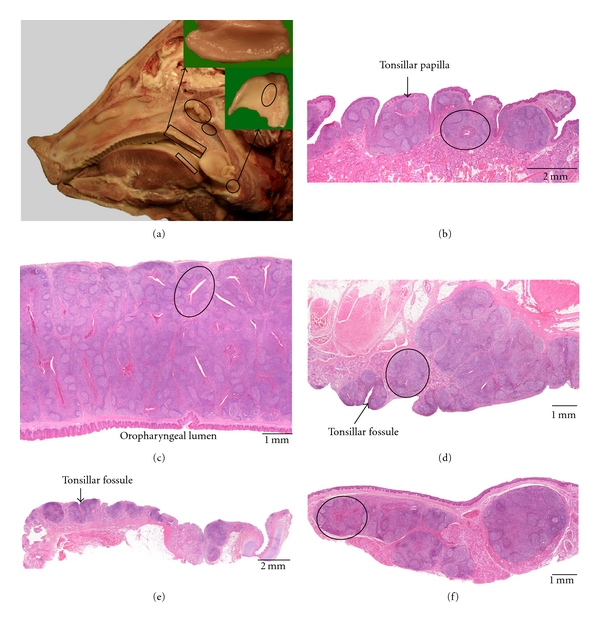
Anatomical localization and histological characteristics of the porcine tonsils. (a) Median section through a porcine head. The pharyngeal tonsil (large oval area) and tubal tonsil (small oval area) in the nasopharynx, the tonsil of the soft palate at the oral side of the soft palate (larger rectangular area, detail shown in upper right insert), and the lingual tonsil (smaller rectangular area) and the paraepiglottic tonsil at the lateral base of the epiglottis (encircled area, detail shown in the lower right insert) are indicated. (b–f) Histological sections through parts of the lingual tonsil (b), the tonsil of the soft palate (c), the pharyngeal tonsil (d), the tubal tonsil (e), and the paraepiglottic tonsil (f), respectively. The encircled areas in (c), (d), and (f) delineate a tonsillar follicle with a central crypt.

**Figure 5:**
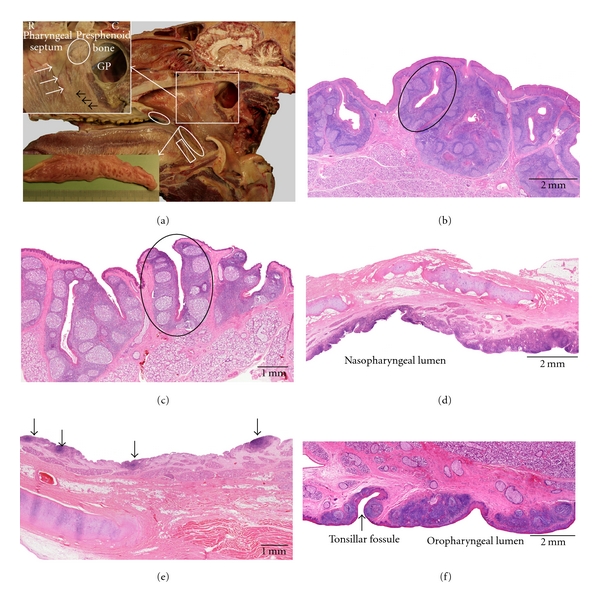
Anatomical localization and histological characteristics of the equine tonsils. (a) Median section through an equine head. The upper left insert shows the pharyngeal and tubal tonsils that are only visible after 4 h fixation in 2% acetic acid. The encircled area in this insert represents the area of the pharyngeal tonsil. Caudoventral to the slit-like opening of the auditory tube (white arrows), numerous white nodules belonging the tubal tonsil (black arrows) can be observed (R: rostral, C: caudal, GP: guttural pouch). The tonsil of the soft palate at the oropharyngeal side of the soft palate (small encircled area), the lingual tonsil (boxed area), and the palatine tonsil (larger encircled area, detail shown in the lower left insert) are additionally illustrated. (b) Histological section through the lingual tonsil. (c–f) Histological sections through parts of the palatine (c), pharyngeal (d), and tubal (e) tonsils and the tonsil of the soft palate (f), respectively. Primary lymphoid follicles belonging to the tubal tonsil are indicated by arrows. Tonsillar follicles are encircled in (b) and (c).

**Figure 6:**
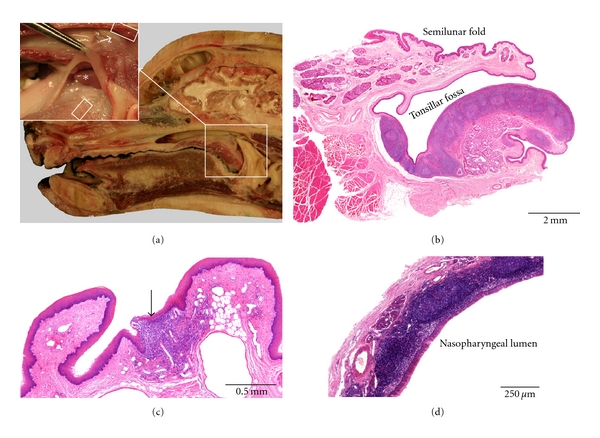
Anatomical localization and histological characteristics of the canine tonsils. (a) Median section through a canine head. The insert shows a higher magnification of the region in which the tonsils are located. In the dog, the lingual (lower boxed area of the insert), palatine (asterisk), and pharyngeal tonsils (upper boxed area of the insert caudodorsal to the opening of the auditory tube (arrow)) are present. (b) Histological section through the palatine tonsil that is partly covered by the semilunar fold. (c) Histological view of a part of the lingual tonsil that is composed of scattered aggregations of lymphoid cells (arrow). (d) Histological section through a part of the pharyngeal tonsil.

**Figure 7:**
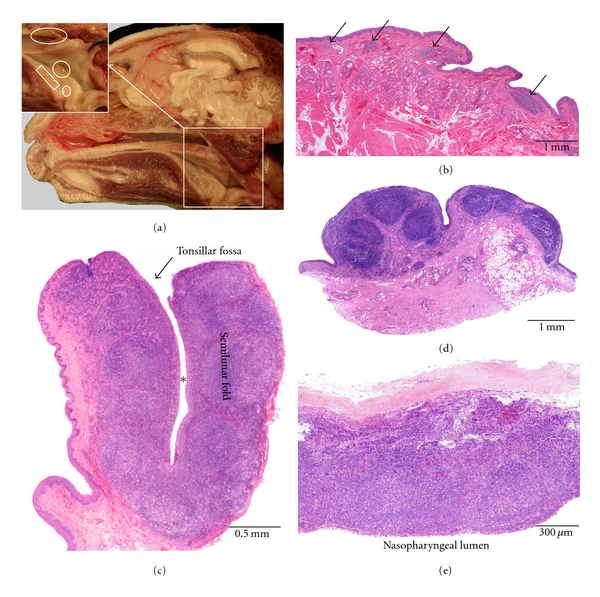
Anatomical localization and histological characteristics of the feline tonsils. (a) Median section through a feline head. The insert shows a larger magnification of the region in which the tonsils are located. The lingual (rectangular box), palatine (larger circle), paraepiglottic (smaller circle), and pharyngeal tonsils (oval) are indicated. (b) Histological section through a part of the lingual tonsil that is composed of scattered aggregations of lymphoid cells (arrows). (c) Histological section through the palatine tonsil which contains a central tonsillar fossa (asterisk). (d) and (e) Histological sections through the paraepiglottic tonsil (d) and a part of the pharyngeal tonsil (e), respectively.

**Figure 8:**
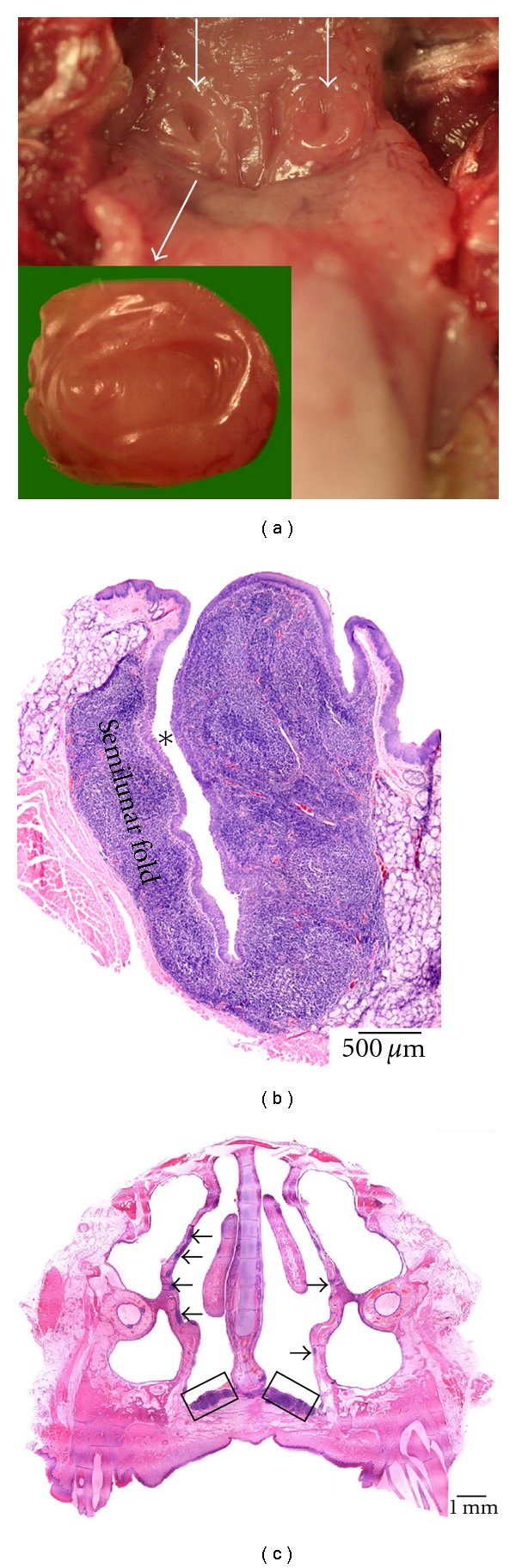
Anatomical localization and histological characteristics of the rabbit tonsils. (a) Rostral view on the paired palatine tonsil (arrows). An excised palatine tonsil is illustrated at higher magnification in the insert. (b) Histological section through a palatine tonsil. The tonsillar fossa is indicated by the asterisk. (c) Histological cross-section through the rabbit nose showing the locations of NALT. Organized lymphoid tissue is present at the bottom of the ventral nasal meatus (boxed areas). Diffuse lymphoid tissue is located along the mucosal linings of the lateral walls of the nasal cavity (arrows).

**Figure 9:**
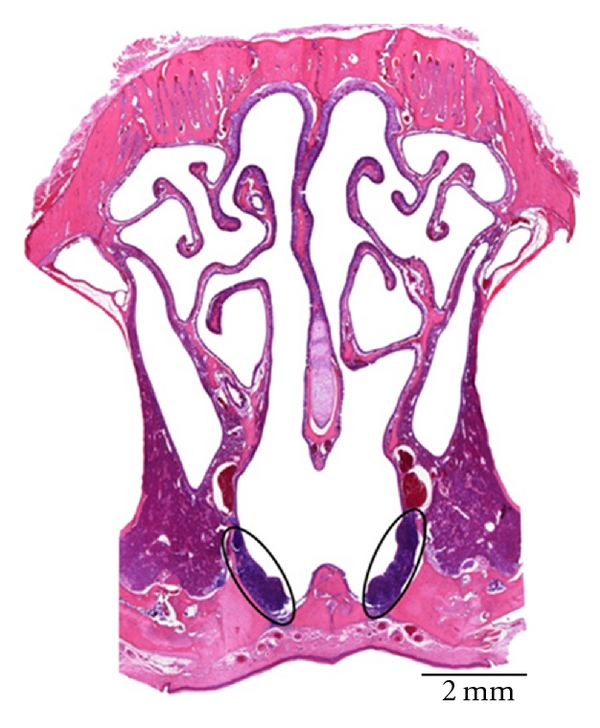
Histological cross section through the nose of a rat. The NALT at the bottom of the ventral nasal meatus is encircled.

**Figure 10:**
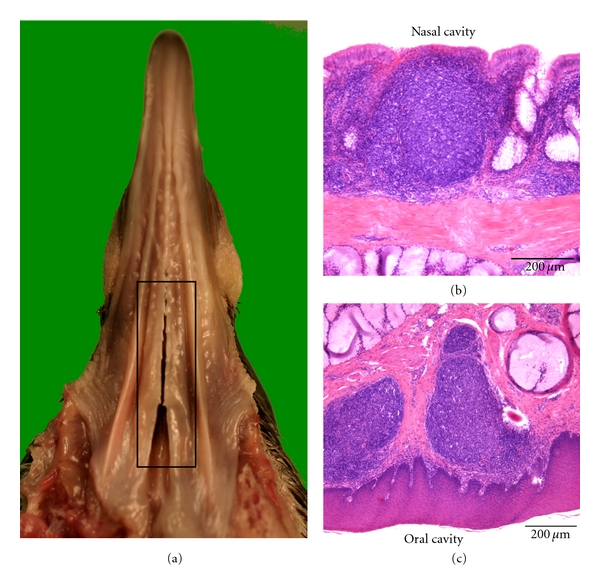
(a) Ventral view of the palate of a pigeon showing the location of the lymphoid tissue around the choanal and infundibular clefts (boxed area). (b) and (c) Histological sections of the palate showing organized lymphoid tissue that is present underneath the respiratory epithelium lining the nasal cavity (b) and underneath the keratinized stratified squamous epithelium lining the oral cavity (c).

**Table 1:** Presence and relative volume of the tonsils and lymphoid tissues in various domestic animals.

	Lingual tonsil	Palatine tonsil	Paraepiglottic tonsil	Pharyngeal tonsil	Tubal tonsil	Tonsil of the soft palate	NALT
Ox	++	+++	−	+++	+	+	
Sheep	±	+++	+	+++	+	+	
Goat	±	+++	±/−	+++	+	+	
Pig	++	−	+	++	+	+++	
Horse	++	+++	−	+	±	++	
Dog	±	+++	−	++	−	−	
Cat	±	+++	+/±	++	−	−	
Rabbit	−	+++	−	−	−	−	++
Rat	−	−	−	−	−	−	+
Pigeon	−	−	−	+	−	−	+

## References

[1] Kuper CF, Koornstra PJ, Hameleers DMH (1992). The role of nasopharyngeal lymphoid tissue. *Immunology Today*.

[2] Strid J, Hourihane J, Kimber I, Callard R, Strobel S (2004). Disruption of the stratum corneum allows potent epicutaneous immunization with protein antigens resulting in a dominant systemic Th2 response. *European Journal of Immunology*.

[3] Liebler-Tenorio EM, Pabst R (2006). MALT structure and function in farm animals. *Veterinary Research*.

[4] Casteleyn C (2010). *Morphological characteristics of the ovine tonsils—a study of their volume and surface area, lymphoid tissue organization, lining epithelia and vascular architecture*.

[5] Vaerman JP, Férin J (1975). Local immunological response in the vagina, cervix and endometrium. *Acta Endocrinologica*.

[6] Brandtzaeg P (2010). Function of mucosa-associated lymphoid tissue in antibody formation. *Immunological Investigations*.

[7] Cesta MF (2006). Normal structure, function, and histology of mucosa-associated lymphoid tissue. *Toxicologic Pathology*.

[8] Quiding-Järbrink M, Granström G, Nordström I, Holmgren J, Czerkinsky C (1995). Induction of compartmentalized B-cell responses in human tonsils. *Infection and Immunity*.

[9] Ogra PL (2000). Mucosal immune response in the ear, nose and throat. *Pediatric Infectious Disease Journal*.

[10] Zuercher AW, Coffin SE, Thurnheer MC, Fundova P, Cebra JJ (2002). Nasal-associated lymphoid tissue is a mucosal inductive site for virus-specific humoral and cellular immune responses. *Journal of Immunology*.

[11] Bienenstock J, McDermott MR (2005). Bronchus- and nasal-associated lymphoid tissues. *Immunological Reviews*.

[12] Harkema JR, Carey SA, Wagner JG (2006). The nose revisited: a brief review of the comparative structure, function, and toxicologic pathology of the nasal epithelium. *Toxicologic Pathology*.

[13] Wu HY, Nikolova EB, Beagley KW, Russell MW (1996). Induction of antibody-secreting cells and T-helper and memory cells in murine nasal lymphoid tissue. *BMC Immunology*.

[14] Zuercher AW (2003). Upper respiratory tract immunity. *Viral Immunology*.

[15] Kiyono H, Fukuyama S (2004). Nalt-versus Peyer’s-patch-mediated mucosal immunity. *Nature Reviews Immunology*.

[16] Cocquyt G, Baten T, Simoens P, Van Den Broeck W (2005). Anatomical localisation and histology of the ovine tonsils. *Veterinary Immunology and Immunopathology*.

[17] von Waldeyer-Hartz W (1884). Ueber den lymphatischen apparat des pharynx. *Deutsche Medizinische Wochenschrift*.

[18] Manesse M, Sautet J, Delverdier M, Schelcher F, Espinasse J, Cabanie P (1995). Anneau de Waldeyer des bovins: anatomie topographique et microscopique des tonsilles. *Revue de Médecine Vétérinaire*.

[19] Perry M, Whyte A (1998). Immunology of the tonsils. *Immunology Today*.

[20] Brandtzaeg P, Bienenstock J (1984). Immune functions of human nasal mucosa and tonsils in health and disease. *Immunology of the Lung and Upper Respiratory Tract*.

[21] Bernstein JM, Gorfien J, Brandtzaeg P, Ogra P (1999). Immunobiology of the tonsils and adenoids. *Mucosal Immunology*.

[22] Cornes JS (1965). Number, size and distribution of Peyer’s patches in the human small intestine. *Gut*.

[23] Casteleyn C, Simoens P, Van den Broeck W (2011). Terminology of the tonsils. *Anatomia Histologia Embryologia*.

[24] World Association of Veterinary Anatomists *Nomina Anatomica Veterinaria, 5th edition*.

[25] Breugelmans S, Casteleyn C, Simoens P, Van den Broeck W Distribution of the lingual lymphoid tissue in domestic ruminants.

[26] Habel RE, Getty R (1975). Ruminant digestive system. *Sisson and Grossman’s The Anatomy of the Domestic Animals*.

[27] Bacha WJ, Bacha LM (2000). *Color Atlas of Veterinary Histology*.

[28] Brandtzaeg P, Kiyono H, Pabst R, Russell MW (2008). Terminology: nomenclature of mucosa-associated lymphoid tissue. *Mucosal Immunology*.

[29] Casteleyn C, Cornillie P, Simoens P, Van den Broeck W (2008). Stereological assessment of the epithelial surface area of the ovine palatine and pharyngeal tonsils. *Anatomia Histologia Embryologia*.

[30] Barone R, Barone R (1997). Pharynx et oesophage. *Anatomie Comparée des Mammifères Domestiques*.

[31] Habel R, Budras K-D, Budras K-D, Habel R (2003). Head. *Bovine Anatomy—An Illustrated Text*.

[32] Ackerknecht E, Zietzschmann O, Ackerknecht E, Grau H (1943). Das Eingeweidesystem. *Ellenberger-Baum—Handbuch der Vergleichenden Anatomie der Haustiere*.

[33] Dyce KM, Sack WO, Wensing CJG (1991). *Anatomie der Haustiere—Lehrbuch für Studium und Praxis*.

[34] Schummer A, Nickel R, Nickel R, Schummer A, Seiferle E (1973). The viscera of the domestic mammals. *Textbook of the Anatomy of the Domestic Animals*.

[35] Habermehl K-H, Frewein J, Vollmerhaus B (1994). Kopfdarm und Gebiß. *Anatomie von Hund und Katze*.

[36] Kraal G, Mestecki J, Lamm ME, Strober W, Bienenstock J, McGhee JR, Mayer L (2004). Nasal-associated lymphoid tissue. *Mucosal Immunology*.

[37] Vollmerhaus B, Friess A, Waibl H, Frewein J, Vollmerhaus B (1994). Imunorgane und Lymphgefäße. *Anatomie von Hund und Katze*.

[38] Berg R (1995). *Angewandte und Topographische Anatomie der Haustiere*.

[39] Salomon F-V, Salomon F-V, Geyer H, Gille U (2005). Verdauungsapparat, apparatus digestorius. *Anatomie für die Tiermedizin*.

[40] Hobson HP (1995). Brachycephalic syndrome. *Seminars in Veterinary Medicine and Surgery (Small Animal)*.

[41] Brandtzaeg P (2003). Immunology of tonsils and adenoids: everything the ENT surgeon needs to know. *International Journal of Pediatric Otorhinolaryngology*.

[42] Pabst R (2007). Plasticity and heterogeneity of lymphoid organs: what are the criteria to call a lymphoid organ primary, secondary or tertiary?. *Immunology Letters*.

[43] Hoquet F, Higgins R, Lessard P, Vrins A, Marcoux M (1985). Comparison of the bacterial and fungal flora in the pharynx of normal horses and horses affected with pharyngitis. *The Canadian Veterinary Journal*.

[44] Wissdorf H, Otto B, Gerhards H, Wissdorf H, Gerhards H, Huskamp B, Deegen E (2002). Schlundkopf, Rachen, Pharynx. *Praxisorientierte Anatomie und Propädeutik des Pferdes*.

